# Longitudinal patterns of physical activity in children aged 8 to 12 years: the LOOK study

**DOI:** 10.1186/1479-5868-10-81

**Published:** 2013-06-21

**Authors:** Rohan M Telford, Richard D Telford, Ross B Cunningham, Thomas Cochrane, Rachel Davey, Gordon Waddington

**Affiliations:** 1 Centre for Research and Action in Public Health, University of Canberra, Bruce ACT 2617, Australia; 2 Medical School, College of Medicine, Biology and Environment, Australian National University, Canberra ACT 0200, Australia; 3 Academic Unit of Internal Medicine, Canberra Hospital, Canberra ACT 2606, Australia; 4 Fenner School of Environment and Society, College of Medicine, Biology and Environment, Australian National University, Canberra ACT 0200, Australia; 5 Faculty of Health, University of Canberra, Bruce ACT 2617, Australia

**Keywords:** Longitudinal, Physical activity, Children, Patterns, Habitual, Recommendations, Trends, Daily, Pedometers, Accelerometers

## Abstract

**Background:**

Data on longitudinal monitoring of daily physical activity (PA) patterns in youth over successive years is scarce but may provide valuable information for intervention strategies aiming to promote PA.

**Methods:**

Participants were 853 children (starting age ~8 years) recruited from 29 Australian elementary schools. Pedometers were worn for a 7-day period each year over 5 consecutive years to assess PA volume (steps per day) and accelerometers were worn concurrently in the final 2 years to assess PA volume (accelerometer counts (AC) per day), moderate and vigorous PA (MVPA), light PA (LPA) and sedentary time (SED). A general linear mixed model was used to examine daily and yearly patterns.

**Results:**

A consistent daily pattern of pedometer step counts, AC, MVPA and LPA emerged during each year, characterised by increases on school days from Monday to Friday followed by a decrease on the weekend. Friday was the most active and Sunday the least active day. The percentage of girls and boys meeting international recommendations of 11,000 and 13,000 steps/day respectively on a Monday, Friday and Sunday were 36%, 50%, 21% for boys and 35%, 45%, 18% for girls. The equivalent percentages meeting the recommended MVPA of >60 min/day on these days were 29%, 39%, 16% for boys and 15%, 21%, 10% for girls. Over the 5 years, boys were more active than girls (mean steps/day of 10,506 vs 8,750; p<0.001) and spent more time in MVPA (mean of 42.8 vs 31.1 min/day; p<0.001). Although there was little evidence of any upward or downward trend in steps/day from age 8 to 12 years, there was a trend toward lower MVPA, LPA and a corresponding increase in SED from age 11 to 12 years.

**Conclusion:**

A weekly pattern of PA occurred in children as young as age 8 on a day by day basis; these patterns persisting through to age 12. In addition to supporting previous evidence of insufficient PA in children, our data, in identifying the level and incidence of insufficiency on each day of the week, may assist in the development of more specific strategies to increase PA in community based children.

## Background

A reported decline in physical activity (PA) among youth [1], coupled with a growing body of evidence supporting the health benefits of PA [2,3], has resulted in an increasing need to understand PA behaviour and patterns among children.

PA among children is often referred to in the literature in terms of ‘patterns’ and is thought to be ‘habitual’ in nature, however the extent to which PA behaviour adheres to patterns or whether these patterns are repetitive to such an extent that they are habitual is not well understood.

Previous cross sectional studies among youth have examined PA patterns in terms of day type (weekday vs weekend) [4-9], school-time versus non school-time [10,11] and time spent in relative exercise intensities [6,12,13]. There is also emerging longitudinal evidence that time spent engaged in moderate to vigorous activity (MVPA) in childhood tracks into adolescence particularly for girls [14] and that daily MVPA and MVPA performed during school breaks (recess and lunch) declines with age [15]. However, there have been no studies incorporating all days of the week using longitudinal data to determine whether specific day of the week habitual patterns or routines of PA might exist or persist in children in the pre-teenage (elementary or primary school) years.

We extend on our previous work with pedometers in 8-10 year-olds [16] by tracking the cohort over 5 years through to age 12 and analysing patterns of PA intensity with the addition of accelerometry. Identification of patterns of activity, in particular those days consistently associated with low or high PA may be important in understanding how interventions are best implemented to reshape the culture of school and weekend days with respect to increasing physical activity in children.

The objective of the current study was to investigate pedometer-assessed PA over 5 consecutive years, with years 4 and 5 supplemented with concurrently assessed accelerometer data, in a large cohort of elementary school children in order to answer the following questions: (a) do children exhibit a general day to day pattern of PA each week? (b) do daily patterns of PA persist each year? i.e. do they change with age? and (c) what percentage of our Australian cohort of midrange socioeconomic status meet currently recommended daily PA levels, and how is this affected by day of the week?

## Methods

### Participants

This study is part of the multidisciplinary Lifestyle of our Kids (LOOK) project [17], which commenced in 2005 with the aim of investigating how early physical activity and physical education contributes to health and development. The overall LOOK study utilizes a longitudinal design and incorporates measures of PA, fitness, motor control, psychological health, family influences, bone health, cardiovascular function, academic achievement and nutrition. The present investigation examines only the patterns of physical activity.

Participants were initially 853 children (435 boys and 418 girls), recruited from 29 government-funded elementary schools in the city of Canberra, Australia. To be included in the study parents provided written consent indicating their child’s acceptance to participate and that their child was able to take part in vigorous physical activity in order to perform fitness tests that were part of the overall study design. Of the children invited to participate, 88% accepted, and represented approximately 30% of the total number of grade 2 children enrolled in Canberra government elementary schools in 2005. Using Australian Taxation Office statistics in 2005, average taxable family income in the suburbs of residence of our cohort approximated the national average. From demographic information obtained from a parent completed questionnaire, approximately 90% of the study participants had one or both parents of Caucasian descent, 7% of Asian descent, 1% indigenous Australian or Polynesian, with 2% unknown. Height and weight was recorded using a stadiometer and digital scales. Means (and standard deviation) of participant characteristics at the time of the first measures of PA were: age 8.0 (0.3) years for both sexes; height 1.31 (0.06) m and 1.30 (0.06) m; weight 29.7 (5.7) and 29.5 (6.5) kg for boys and girls respectively.

Of the 853 children who gave consent to participate in baseline measurements at age 8 years, 640 (75%) participated in the final year of measurement at age 12 years. Study attrition over the four years of measurement was due to children moving to a school outside the study jurisdiction, including interstate (n=193), or withdrawing from the study (n=20).

This study was approved by the Australian Capital Territory Health and Community Care Human Research Ethics Committee and the Ethics Committee of the Australian Institute of Sport. Participation by the children was entirely voluntary and informed consent was received from all the parents or guardians.

#### Pedometry

We used pedometers of identical step detection mechanism throughout the study, distributed by Walk 4 Life (Plainfield, IL USA) in the first year, and by New Lifestyle, A-82 (Lee's Summit, MO USA) in ensuing years. The change was due to the latter model having an identical step count mechanism with additional 7 day memory which obviated the need for daily recordings. The two models of pedometer have previously been shown to provide equivalent step counts [16] and the validity and reliability of the pedometer mechanism has been verified with children of about the same age as our cohort [18]. Prior to pedometers being issued to children each year, a routine calibration check was carried out on every pedometer by a technician who performed a 40 step trial with the pedometer located on the right hip and only pedometers that were accurate to within 0.5% were used.

Pedometers were issued to the children for one week on five separate occasions one year apart. The testing periods were confined to the same period in each year, in the Australian Spring to early Summer months of September to December in 2005, 2006, 2007, 2008 and 2009 and included a weekend. The weekdays were normal school days. Children wore pedometers on their right hip for seven consecutive days, and the first day’s measurement was randomly distributed from Monday through to Friday. The first day’s measurement was not included in our analysis given that part of it was incomplete, but also because the novelty of wearing the pedometers may have influenced initial activity. Pedometer step counts were routinely recorded by teachers and parents in the first year and by a technician in the remaining years. As previously suggested [8], daily step counts less than 1000 and greater than 30,000, were considered erroneous and discarded. In order to maximise data, all valid days were used in the analyses, our statistical model adjusting for incomplete data sets.

#### Accelerometry

In the final two years of elementary school (grades 5 and 6), children’s activity was also assessed using accelerometers (Actigraph GT1M, Pensacola, FL, USA). Accelerometers were positioned on a belt around the waist in line with the right knee. Daily accelerometer counts (AC) were used as a measure of volume of PA and cut off points for sedentary classified activity (SED) were defined as 0-100 counts per minute, light physical activity (LPA) defined as 101 – 2,296 counts per minute and moderate to vigorous physical activity (MVPA) classified as greater than 2,297 counts per minute based on published recommendations [19,20]. An epoch length of 60 seconds was used and the first day’s data were discarded to minimize any potential reactivity. Days of accelerometer data were included if there were 10 or more hours of activity; and in the absence of any established non-wear time criteria for children in the literature [21], the decision was made to consider an entire hour to be invalid if there were more than 30 zero counts in a row (30 minutes of non-wear time). For comparisons of PA between school time and outside of school time, school hours was considered to be between 9 am and 3 pm and outside of school hours was the sum of before school (7 am-9 am) and after school (3 pm–10 pm). For this analysis, days of accelerometer data were included if there were more than 10 hours of activity and data were recorded for each time period. Actigraph accelerometers have been validated for use in both children and adolescents [22]. Accelerometer data were analyzed without the low frequency extension (LFE) function using Meterplus software, Version 4.2 (San Diego State University, USA).

### Statistical analysis

Our data are multi-level and the response variables representing physical activity vary at a between-subject and at two temporal levels; the temporal levels consist of five separate years for pedometer data (two separate years for accelerometer data) and, within years (seven observations corresponding to each day of the week). We specify subject, year-within subject and day-within-year-within subject as random effects in our formulated statistical model to account for possible dependencies in observations due to the above multi-level structure. As our main focus was on patterns of systematic variation between genders, between grades, between day of the week and possible interaction between these factors, these terms were specified in our model as fixed effects. Post hoc analysis introducing wear time into our model was performed to examine any potential influence of wear time on these patterns. For analysis of aggregate school-time/non-school time data, the same modelling framework was adopted. In this case, however, our model does not include random and fixed terms which include day of week. Our statistical model then fits within the general framework of general linear mixed models [23]. Restricted maximum likelihood is used to estimate variance components and weighted least squares for estimating fixed effects. Statistical significance of effects are assessed by calculating adjusted Wald statistics [24].

Preliminary analysis showed pedometer and accelerometer data was positively skewed so a square root transformation was used for both accelerometer and pedometer data to satisfy linearity as well as distributional assumptions of our model. General model checking procedures were routinely used to identify aberrant data and to check the model assumptions. Adjustment for incomplete data and missing values were accommodated for in the estimation process and so all valid days of data for all children were utilised. Statistical computations were undertaken using the statistical package Genstat, Version 14 (VSN International Ltd, Oxford, UK).

## Results

### Pedometer step counts

The percentages of children who were issued devices and returned valid pedometer data in each measurement year are shown in Table 1.

**Table 1:** Descriptive statistics for pedometer measured physical activity for each measurement year separated by gender

	**2005**		**2006**		**2007**		**2008**		**2009**	
	**Grade 2**		**Grade 3**		**Grade 4**		**Grade 5**		**Grade 6**	
	**Age 8 years**		**Age 9 years**		**Age 10 years**		**Age 11 years**		**Age 12 years**	
	**Boys**	**Girls**	**Boys**	**Girls**	**Boys**	**Girls**	**Boys**	**Girls**	**Boys**	**Girls**
**N pedometers issued**	435	418	397	383	347	336	330	314	328	312
**N (%) returned pedometers meeting validation criteria**	372 (86%)	366 (88%)	290 (73%)	308 (80%)	285 (82%)	283 (84%)	272 (82%)	256 (82%)	266 (81%)	274 (%88)
**Steps per day** (median and 5^th^ and 95^th^ percentiles)	12014 (4264,21303)	9762 (3259,18195)	10552 (2059,20933)	8523 (2126,16541)	11206 (2890,21171)	9078 (2495,16868)	11459 (3143,20705)	10002 (3017,17647)	10477 (2329,19244)	9008 (2628,15947)
**% meeting step per day recommendations **^**a**^	42	37	34	30	38	33	39	38	30	30

^a^ Step recommendations based on accumulating >11,000 steps/day for girls and >13,000 steps/day for boys.

As a square root transform was applied to each PA variable in our analyses to meet assumptions of normality, values in text are back-transformed and presented as geometric means. Figures contain transformed data and descriptive data are presented as medians with 5^th^ and 95^th^ percentiles in tables.

#### Year to year and gender variation in step counts

**Figure 1:**
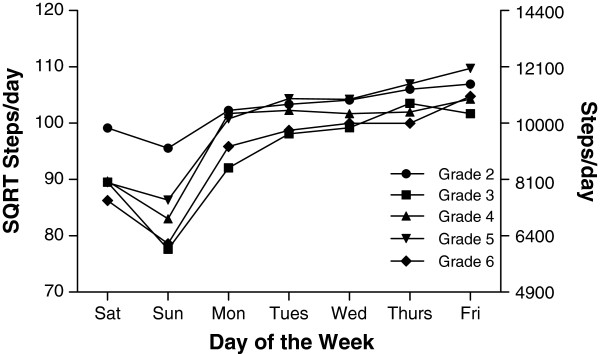
**Mean pedometer steps per day, girls and boys combined for each measurement year from grade 2 (age 8 years) to grade 6 (age 12 years).** The left Y axis represents the square root of steps per day used for statistical analysis and the non linear right Y axis represents actual steps per day.

Pedometer data for each measurement year are shown in Table 1. There were significant year to year variations in daily step counts (p<0.001), but there was no evidence of any consistent trend towards an increase or decline in PA (Figure 1). The number of steps/day declined for both boys and girls between 2005 and 2006, increased from 2006 to 2008, followed by another decline from 2008 to 2009. No significant year by gender interactions were found. Combining data from all years, boys were generally more physically active than girls, boys averaging 10,506 steps/day vs. girls 8,750 steps/day (p<0.001).

#### Day of the week and gender variation in step counts

**Figure 2:**
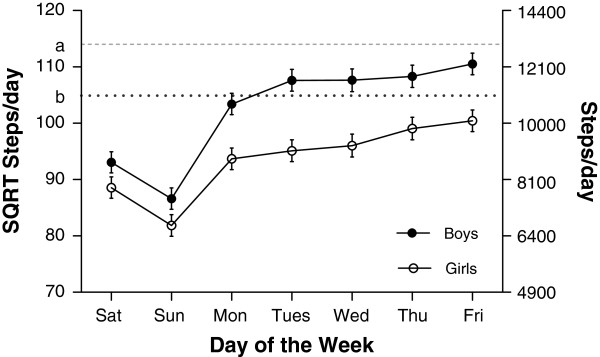
**Mean (and 95% confidence intervals) pedometer derived steps per day for all 5 measurement years combined separated by gender.** Differences were found for step counts according to day of the week for both boys and girls (both p<0.001). Overall, boys took more steps per day than girls (p<0.001). The horizontal lines a and b represent published step per day recommendations of 13,000 steps for boys and 11,000 steps/day for girls respectively.

Median values for steps taken on each day of the week are shown in Table 2. Our model indicated that the number of steps/day varied according to day of the week (boys and girls both p<0.001). To avoid statistical issues associated with multiple post-hoc pair-wise testing we refer to the figures and tables to provide an indication of the day to day differences. Figure 2 shows the day to day variation in mean step counts for boys and girls for the five measurement years combined. For both boys and girls, there was a trend of an increase in daily step counts from Monday through to Friday, the difference between Monday and Friday being 12% (1,524 steps/day) for boys and 13% (1,312 steps/day) for girls. This was followed by a decrease from Friday through to Sunday, with the difference between Friday and Sunday being 36% (4,714 steps/day) for boys and 34% (3,393 steps/day) for girls. Sunday was the least active day of the week. There were clear gender differences, boys took significantly more steps/day than girls on all days of the week (p<0.001) and this difference was greater on school days than on weekend days. For example, on Wednesday, the difference in mean steps/day between boys and girls was 20% (2,360 steps/day) compared with a difference of 15% (806 steps/day) on Sunday.

**Table 2:** **Unadjusted median values with 5**^**th **^**and 95**^**th **^**percentiles for physical activity measured by pedometers and accelerometers categorised by gender and day of the week for all measurement years combined**

	**Mon**		**Tues**		**Wed**		**Thurs**		**Fri**		**Sat**		**Sun**	
	**Boys**	**Girls**	**Boys**	**Girls**	**Boys**	**Girls**	**Boys**	**Girls**	**Boys**	**Girls**	**Boys**	**Girls**	**Boys**	**Girls**
**Pedometer steps/day**	11550	9409	12276	9442	12104	9995	12204	10476	12971	10392	9134	8338	8008	6941
	(3774-19545)	(3031-16906)	(4114-21412)	(3141-16384)	(3627-20841)	(2808-17325)	(4106-21379)	(3750-17296)	(3505-22049)	(3451-18751)	(2189-20033)	(2099-16984)	(1852-18418)	(1919-15620)
**% meeting step per day recommendations **^**a**^	36	32	43	34	44	38	44	44	50	45	26	28	21	18
**Accelerometer wear time (hr/day, mean and standard deviation)**	13.16	13.16	13.19	13.18	13.05	13.29	13.06	13.26	13.88	13.98	13.08	13.22	12.57	12.35
	(1.74)	(1.58)	(4.6)	(4.5)	(4.52)	(1.44)	(1.61)	(1.5)	(1.75)	(1.71)	(1.86)	(1.84)	(1.74)	(1.71)
**% returned accelerometers meeting validation criteria **^**c**^	70	70	71	70	70	75	72	70	73	74	60	62	53	54
**Accelerometer Counts (counts per day/1000)**	397	338	419	361	434	363	447	387	457	407	338	316	307	302
	(210-807)	(189-663)	(216-885)	(184-656)	(250-869)	(204-754)	(238-849)	(206-707)	(254-830)	(233-727)	(144-796)	(148-795)	(123-796)	(122-763)
**MVPA (min/day)**	42	30	44	32	49	33	52	35	51	37	27	22	23	18
	(11-104)	(7-85)	(11-121)	(6-76)	(13-117)	(7-85)	(11-117)	(7-87)	(15-118)	(10-95)	(3-90)	(2-81)	(2-90)	(2-78)
**Light (min/day)**	339	340	340.5	340	351	341	338	354	369.5	376	338	342	315	316
	(231-466)	(231-440)	(133-468)	(184-472)	(228-459)	(224-440)	(221-469)	(234-466)	(247-488)	(256-492)	(192-447)	(210-494)	(180-447)	(188-457)
**Sedentary (min/day)**	385	408	369	390	372	400	367	389	380	391	385	381	380	371
	(250-534)	(275-536)	(178-517)	(209-527)	(231-501)	(271-552)	(240-505)	(244-536)	(243-528)	(253-537)	(226-566)	(233-549)	(233-566)	(226-530)
**% meeting MVPA recommendations **^**b**^	29	15	33	16	38	18	41	19	39	21	22	12	16	10

^a^ Step recommendations based on accumulating >11,000 steps/day for girls and >13,000 steps/day for boys.

^b^ MVPA recommendations based on accumulating >60 minutes of MVPA per day.

^c^ Validation criteria based on >10 hours of wear time per day.

#### Longitudinal persistence of a day of the week step pattern

Our longitudinal analysis indicated that day of the week step patterns persisted from age 8 to 12 years. As shown in Figure 1, while the volume of steps fluctuated between years (p<0.001) there is evidence of a persistent year to year pattern characterised by an accumulation of steps/day during the school week followed by a progressive decline on weekends.

### Accelerometers

Eighty five per cent of children in grade 5 and 84% in grade 6 returned accelerometers with valid data and were included in analyses. Differences were found in wear time according to day of the week (p<0.001). For boys and girls combined, wear time was highest on Fridays (13.92 h SD=1.75) and lowest on Sundays (12.48 h SD=1.74). No significant differences in wear time were found between boys and girls (p>0.3). The compliance of children returning valid data according to day of the week is shown in Table 2. Compliance was found to be lowest on weekend days.

Descriptive data for accelerometer variables for each measurement year are shown in Table 3. Overall boys accumulated 12% more AC per day than girls (boys 413,000, girls 364,000 counts/day, p<0.001), and spent 25% more time in MVPA (boys 42.8, girls 32.1 min/day, p<0.001). No significant difference was found between boys and girls for time spent in LPA per day (boys 342, girls 348 min/day, p=0.22) and girls spent 3% more minutes per day in SED classified activities than boys (boys 384, girls 396 min/day; p=0.013). Patterns were similar to those for the pedometer data; gender differences for AC and time spent in MVPA were greater on school days than weekends. For example, on Wednesday boys participated in 33% more MVPA (17.1 min/day) compared to Sunday where the difference was 15% (3.9 min/day).

**Table 3:** **Unadjusted Median values with 5**^**th **^**and 95**^**th **^**percentiles for accelerometer measured physical activity for each measurement year separated by gender**

	**2008**		**2009**	
	**Grade 5**		**Grade 6**	
	**Age 11.1±0.4 y**		**Age 12.1±0.4 y**	
	**Boys**	**Girls**	**Boys**	**Girls**
**N accelerometers issued**	330	314	328	312
**N (%) returned accelerometers meeting validation criteria**	282	266	270	265
	(85%)	(85%)	(82%)	(85%)
**Accelerometer wear time (hrs per day)**	13	13	13	13
	(10,17)	(10,17)	(10,17)	(10,17)
**Accelerometer Counts (counts per day/1000)**	418	375	390	342
	(196,663)	(183,789)	(159,782)	(157,642)
**MVPA (min/day)**	43 (6,116)	31 (5,88)	43 (6,110)	30 (4,81)
**Light (min/day)**	355	359	333	333
	(223,474)	(232,488)	(199,465)	(213,449)
**Sedentary (min/day)**	364	376	388	406
	(220,508)	(228,523)	(252,545)	(259,548)
**% meeting MVPA recommendations **^**a**^	33	18	30	14

*MVPA* moderate and vigorous physical activity.

^a^ MVPA recommendations based on accumulating >60 minutes of MVPA per day.

#### Year to year and gender differences in Accelerometer measures

For both boys and girls there was a decline in AC, MVPA and LPA (all p<0.001) from age 11 to 12 years as shown in Table 3. These decreases corresponded to a decline in MVPA of 4% (1.7 min/day) for boys and 10% (3.0 min/day) for girls, and a decline in LPA of 6% (22 min/day) for boys and 7% (24 min/day) for girls. There was a corresponding increase in time spent in SED from age 11 to 12 (boys and girls both p<0.001) in the order of 6% (24 min/day) for boys and 10% (40 min/day) for girls.

#### Day of the week variations in accelerometer measures

**Figure 3:**
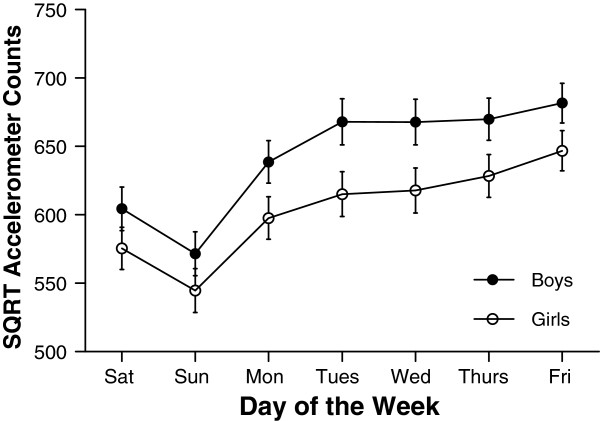
**Mean accelerometer counts per day (and 95% confidence intervals) for each day of the week separated by gender.** Differences were found for accelerometer counts according to day of the week for both boys and girls (both p<0.001). Overall, boys accumulated more accelerometer counts per day than girls (p<0.001).

**Figure 4:**
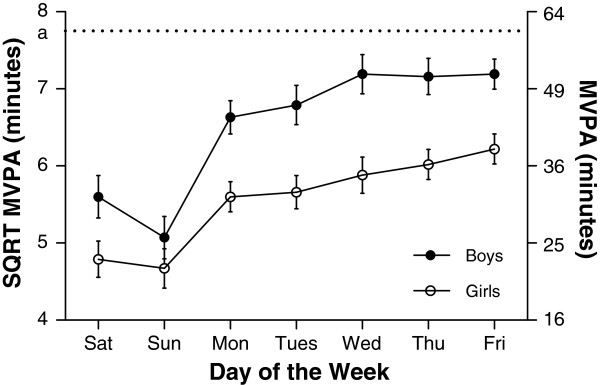
**Mean minutes of moderate and vigorous activity (MVPA) (and 95% confidence intervals) for each day of the week separated by gender.** Differences were found for MVPA according to day of the week for both boys and girls (both p<0.001). Overall boys spent more time in MVPA than girls (p<0.001). The horizontal line a represents the published recommended level of 60 mins MVPA per day.

**Figure 5:**
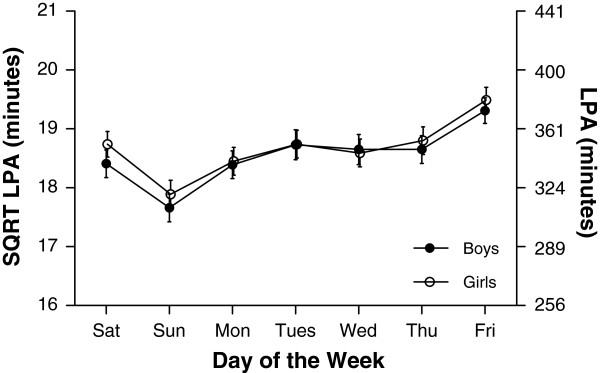
**Mean minutes of light physical activity (LPA) (and 95% confidence intervals) for each day of the week separated by gender.** Differences were found for LPA according to day of the week for both boys and girls (both p<0.001). Overall no differences were found for LPA between boys and girls (p=0.2)

**Figure 6:**
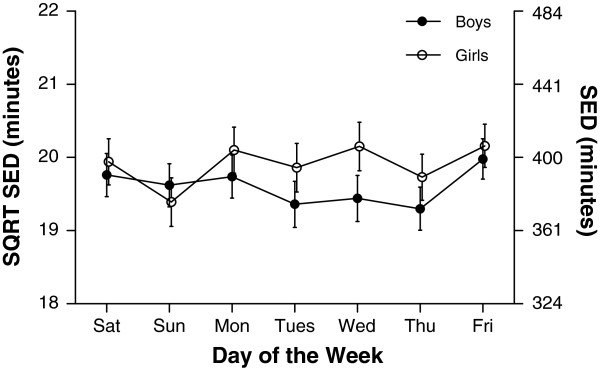
**Mean minutes of sedentary physical activity (SED) (and 95% confidence intervals) for each day of the week separated by gender.** Differences were found for SED according to day of the week for both boys and girls (both p<0.001). Overall girls accumulated more SED per day than boys (p<0.001).

Median values for accelerometer variables for each day of the week are shown in Table 2. Similar to findings from pedometer data, and as shown in Figures 3, 4 and 5, variation occurred in each of AC, MVPA and LPA according to day of the week (all p<0.001), a similar pattern emerging for each PA measure. This was characterised by an increase in PA during school days. For example, time spent in MVPA was 7.7 min/day and 7.3 min/day higher on Friday compared to Monday for boys and girls respectively. Following a rise in MVPA over schooldays was a decrease over the weekend; boys spent 26.0 min per day and girls 16.9 min per day less time in MVPA on Sunday compared to Friday. In contrast with the general pattern evident for AC, MVPA, and LPA, the daily pattern for SED (Figure 6) showed no consistent pattern, fluctuating throughout the week. Post hoc analysis introducing wear time into our model showed no change in any of the patterns observed.

#### PA intensity in and out of school time

Descriptive data for PA intensity in school hours (9 am-3 pm) and outside of school hours (7 am-9 am and 3 pm-10 pm) is shown in Table 4. Both boys and girls spent more time in SED and LPA classified intensity outside of school time compared to during school time. Outside of school time boys spent 38 minutes more in SED classified activity and 42 minutes more in LPA compared to school time (both p<0.001). Girls spent 25 minutes more in SED classified activity and 24 minutes more in LPA outside of school time compared to school time (both p<0.001). For MVPA, in the boys there was no significant difference in MVPA accumulated within and outside school time (23.6 vs 23.2 min; p=0.47). Girls however spent more time engaged in MVPA outside of school time than in school time (18.4 v 14.7 min; p<0.001).

**Table 4:** **Unadjusted medians with 5**^**th **^**and 95**^**th **^**percentiles for sedentary, light and moderate and vigorous activity (MVPA) for school time and non-school time, separated by gender**

	**School Time**	**Non-School Time**
**Sedentary (min)**		
Boys	166 (87-238)	209 (94-330)
Girls	179 (89-250)	215 (106-323)
**Light (min)**		
Boys	160 (84-228)	182 (88-277)
Girls	154 (85-221)	191 (94-283)
**MVPA (min)**		
Boys	20 (2-58)	19 (2-71)
Girls	13 (2-44)	16 (2-58)

#### Comparison with international PA recommendations

Overall, 37% of boys and 34% of girls met published PA recommendations [25] of 11,000 steps per day for girls and 13,000 steps per day for boys. This varied with day of the week, 36% of boys and 35% of girls meeting recommendations on a Monday, 50% boys and 45% girls on Friday and 18% and 21% on a Sunday.

Overall, 31% of boys and 16% of girls met published MVPA recommendations [26] of 60 min per day. Again, the percentages varied with day of the week. Twenty nine percent of boys and 15% of girls performed at least 60 min MVPA on a Monday compared to 39% of boys and 21% of girls and on Friday and only 17% of boys and 10% of girls met recommendations on a Sunday.

## Discussion

The most salient aspects of this novel longitudinal study of pre-teenage children were (a) the finding of a distinct weekly pattern of habitual PA and (b) this pattern was not simply a one off occurrence but persisted for five consecutive years in 8 to 12 year old children. This pattern was characterized by a gradual build-up of daily PA through the school week followed by a progressive drop over the weekend, and extends on previously reported data comparing weekdays and weekends [4-9]. As well as highlighting the general world-wide concern that children are insufficiently physically active, our data indicate that the incidence of children failing to meet recommended daily PA standards varies markedly according to day of the week.

### Day of the week patterns of PA

A large reduction in weekend activity in our cohort, consistent with previous reports [5,9] along with the variation during the weekdays raises the question as to why these day to day variations occur. Our study was not designed to determine causes of the habitual PA patterns observed in children in our cohort, but we might speculate that these occur as a result of socio-cultural influences that surround the working week common to Western culture. Children are introduced to a rigid habitual 7 day weekly cycle composed of 5 days of school followed by two rest/free days as early as the age of 4 years in Australia, and day to day behavioural patterns may exist in line with this culture. Studies examining changes in behaviour according to day of the week are sparse, however there is some support that mood in adults is least positive on Monday (‘Blue Monday’) and improves over the course of the week with a positive boost on Friday (‘Thank God it’s Friday’) [27]. While the idea that mood changes according to day of the week is far from conclusive, it is possible that children in this study may be adhering to underlying day to day societal trends in terms of habitual physical activity. Intervention efforts aimed at increasing PA may need to overcome daily habits that have been formed early in a child’s life and could profit from addressing cultural contingencies surrounding the concept of the working week. There have been various suggestions as to how many days of objectively measured data are enough to adequately reflect PA [28,29]. The day to day variation in our data suggests that the full seven days may be needed to provide a realistic measure of habitual activity. Even taking one weekend day would introduce error given the variation seen in PA performed on Saturday and Sunday. Similarly, exclusion of just one day, particularly Monday or Friday may result in different outcomes.

There is also the question of how best to utilize these data in strategies to increase PA in children. One interpretation of our data might be that school based PA interventions should target days early in the week to increase weekly PA, and this may involve targeting the teachers and their influence on their pupils’ PA early in the week. On the other hand the large discrepancy between weekday and weekend MVPA might indicate the need to target the parents, together with sports coaches and other adult direct influences on PA of the children in the community and home settings.

### Influence of wear time and compliance

Our primary objective was to investigate total daily PA and how this varied according to day of the week. Consequently, and considering that we discarded potentially invalid data, both on an hourly and daily basis, our interest lay mainly with the summation of accelerometer output during the entirety of the day between wake and sleep. However, given that wear time has been shown to influence hourly accelerometer outputs, particularly time spent in sedentary activities in adults [30] it was prudent to introduce wear time into our model to determine whether this variable had any influence on the nature of the physical activity patterns. The absence of any discernible effect after adjusting our model for wear time on the patterns showed, in effect, that if all our participants had equal wear time during the day, the patterns of PA we describe persist.

Corresponding with lower wear time on weekends was the finding of lower accelerometer compliance (fewer children recording a valid day) on these days. While we cannot rule out that compliance influenced daily PA we found no evidence of bias; there was no difference in average daily PA during the week between children who were fully compliant on weekends compared to children who were not (data not shown).

### In-school and out of school time

There are mixed findings in the literature comparing activity levels during school hours and nonschool hours. For example, one study indicated higher levels outside of school [31]. While another showed higher levels during school [32]. In the current study, both boys and girls accumulated more SED and LPA outside of school time than during school time. Our findings are not entirely consistent with findings from a study conducted in 1568 UK 9-10 year old children where it was reported that SED activity was greater outside of school hours for boys but not girls and that both boys and girls performed more vigorous PA outside of school hours than during school [31]. While boys in our cohort accumulated similar amounts of MVPA during and outside of school, girls accumulated more MVPA outside of school hours than in school time. This finding suggests that the girls did not respond to opportunities to be active during school hours as well as boys and supports the premise that different intervention strategies may be required for boys and girls in the school setting [33,34].

### Longitudinal trends

Our data show that the volume of steps taken per day fluctuated from year to year and that there was no evidence of either a systematic increase or decline in volume of PA from age 8 to 12 years. These findings highlight the importance of longitudinal studies carried out over several continuous years in understanding longitudinal trends in PA among children. For example, if we were to only compare data collected in the first and last year of pedometer measures, we may have concluded that PA declined with age. However, if we compared the second and last year we might have concluded that there were no changes in PA. The accelerometer variables AC, MVPA and LPA all decreased and SED increased from age 11-12 years. While pedometer steps/day decreased over the same period, given the fluctuations seen in annual step counts and our lack of accelerometer measures at baseline, it remains unclear whether this provides evidence of a decline in PA as our cohort approached adolescence. While these findings provide support for previous longitudinal studies showing a decline in PA with age in children [15,35], further PA monitoring of our cohort is necessary to answer this question.

Of considerable interest is that the day of the week pattern of PA that existed in our cohort at 8 years was not unique to this age group but persisted in every year of our study through to age 12 years. To our knowledge this is the first study to demonstrate not only a day to day pattern of PA, but also to show that this pattern is consistent in pre-adolescence. Knowledge that persistent patterns exist in elementary school children provides information that can be well utilised in designing interventions to increase PA among youth.

### International recommendations

While comparison to other pedometer studies may be difficult due to cross-population differences in measurements and procedures [36], a recent review concluded that elementary school children should be directed to accumulate 13,000-15,000 steps/day for boys and 11,000-12,000 steps/day for girls to achieve health benefits [25]. Based on these recommendations only 33% boys and 34% girls in our study met these suggested step targets. Of particular concern were the low weekend step counts in our cohort, with only 21% of boys and 18% of girls meeting step count recommendations on a Sunday. Low weekend activity is consistent with previous literature [4-9] and we add our concerns regarding the PA opportunities available to children on the weekend. Our findings extend this concern to schooldays during the week. During school days, only 36% of boys and 35% of girls met stepping recommendations on a Monday compared to 50% for boys and 45% of girls on a Friday, and as suggested above, intervention strategies could benefit from targeting days early in the school week.

Similar concerns for the health of our children are raised by the accelerometer data. Our data add a day by day specificity to the incidence of children failing to reach recommended standards. Current guidelines based on previous research and expert opinion suggest that at least 60 minutes of MVPA per day is required in youth for optimal health benefits [26]. In our cohort only 31% of boys and 16% of girls met these recommendations. Twenty-nine percent of boys and 15% of girls met recommendations on a Monday, compared to 39% of boys and 21% of girls on a Friday and 16% of boys and 10% of girls on a Sunday. Previous studies have reported a large variation in the percent of children reaching recommended MVPA targets [37]. For example, in a large sample of 12 year old children in the UK, only 4.1% of boys and 0.9% of girls were reported as being sufficiently active [6]. Alternatively, in a cohort of 6-11 year-old children in the USA, 48.9% of boys and 34.7% of girls surpassed 60 minutes of MVPA/day [38]. In terms of time spent in MVPA our findings of 43 and 32 min per day for boys and girls respectively are slightly higher than a recent pooled analysis of 20,871 children aged 4-18 years [39] where reported values for MVPA were 37 min per day for boys and 24 min per day for girls. It should be noted that comparisons of accelerometer results have to be interpreted with some caution, due to variation in methodology adopted by researchers to capture and process accelerometer data such as wear time, valid day and cut-point decisions [21]. It has also been suggested that larger epoch lengths may under-report moderate and vigorous activity and that differing epoch lengths should not be compared [40].

### Strengths and weaknesses

A strong aspect of our study was the substantial size of the cohort and the repeated objective measurements of physical activity over 5 successive years in the same months of the year in a city with consistent weather conditions conducive to outside PA. On the other hand our cohort was limited mainly to children in a medium sized city of relatively homogeneous and mid-range socioeconomic status and may not be generalizable to children of other ethnicities and environments. Furthermore our objective measures of activity do not accurately measure non-ambulatory activities such as swimming and cycling, and our data are dependent on children wearing the pedometers and accelerometers as instructed. Even so, the sensitivity and reliability of our procedures were supported by their detection of daily and yearly patterns in our group. In comparing during and outside of school PA, given the general consistency of bell times of government schools in the region we determined school hours as 9 am – 3 pm, however we acknowledge that small variations on bell times may have occurred. Finally it is re-emphasized that our work was confined to the same season of the year. PA has been reported to be season dependent [6,41], and so the pattern which emerged in the spring in our study may not apply to other seasons of the year.

## Conclusion

Our longitudinal data collected for 7 days each year over 5 consecutive years revealed a distinct day to day habitual physical activity pattern. Furthermore this pattern was found to persist from age 8 through to age 12 years. The pedometer assessed volume of PA generally increased on school days from Monday through to Friday before declining dramatically over the weekend, and this pattern also applied to the accelerometer assessed volumes and intensity of PA in the last two years. This new knowledge of day of the week patterns may assist public health strategists in designing more specific interventions to increase PA levels in children.

## Competing interests

The authors declare that they have no competing interests.

## Authors’ contributions

RMT participated in the design and coordination of the study, collected all data and drafted the manuscript. RC performed the statistical analysis. RDT, TC, RD and GW assisted with interpretation and drafting of the manuscript. All authors read and approved the final manuscript.
